# Taking alcohol by deception II: Paraga (alcoholic herbal mixture) use among commercial motor drivers in a south-western Nigerian city

**DOI:** 10.1186/1756-0500-5-301

**Published:** 2012-06-18

**Authors:** Oluwadiya S Kehinde, Fatoye Femi Olusegun

**Affiliations:** 1 Department of Surgery, College of Medicine, Ekiti State University, Ado-Ekiti, Ekiti State, Nigeria; 2 Department of Mental Health, College of Health Sciences, Obafemi Awolowo University, Ile-Ife, Osun State, Nigeria

## Abstract

**Background:**

Paraga, an alcoholic herbal preparation that comes in different varieties had been shown to be commonly available to commercial drivers in southern Nigeria. This study aims to determine the prevalence and pattern of paraga use, and to evaluate the level of awareness of the risks entailed in taking paraga among intercity commercial drivers operating out of motor parks in Osogbo, southwest Nigeria. We administered a locally validated version of the WHO drug and alcohol survey questionnaire to 350 commercial drivers.

**Results:**

Of the 350 questionnaires administered, 332 were used for the data analysis; the remaining 18 were rejected because they had too many missing data. The prevalence rate in the past one year was 53.6% and 43.2% for the past one month (current). Three-quarters were moderate to heavy users, and many take the drug while working. A total of 25.6% had been involved in road crashes after taking paraga and 36.7% had actually seen people getting drunk from taking paraga. Only 40% of the drivers thought paraga use was harmful to their health, the others believing it to have therapeutic values (25%) or undecided (35.0%). Only 43.8% of the drivers would be willing to stop taking paraga.

**Conclusions:**

Paraga use is popular among commercial drivers. Because of its alcoholic nature, drivers’ access to the concoction should be controlled and appropriate enforcement put in place.

## Background

For thousands of years, the use of indigenous plants has played an important role in the treatment of a variety of disorders in the world [1]. An estimated 80% of the developing world population utilizes traditional methods of healing which include herbal remedies [2]. In Nigeria, more than 70% of the population depends on traditional remedies for the initial treatment of diseases and injuries [3,4].

Traditionally, such treatments were handled by medicine men who used a varieties of methods such as medicinal herbs, incantations, rituals, manipulations, splints and divinations to treat their patients [5,6]. However, urbanization has changed this tradition. A new group of itinerant traditional drug peddlers have begun peddling their wares in urban centres in Nigeria. One of the most common herbal preparations being sold is paraga, which has been defined by Oshodi et al. as “a mixture of unrefined or poorly refined alcohol and herbs which is periodically ingested, as a form of self-medication against certain illnesses” [7]. A study among secondary school students in Lagos showed that 51.5% of them have ingested paraga in the past [7]. Its use is said to be common among drivers and there have been numerous anecdotal and newspaper reports that as well as alcohol, paraga makers sometimes include psychoactive herbs such as cannabis and cocaine in paraga to add some “kick” to their products [8]. There have been concerns by safety experts about the synergetic effects that drugs and alcohol may have on each other when combined together [9]. For example, cannabis and alcohol in combination carry a potential greater risk than either of them alone [9,10].

Paraga use is believed to be common among drivers, but to our knowledge, no study on its use has been conducted among them. This study is an attempt to fill the gap in the literature. It is a three-part study. The aim of the first part was to determine the ethanol concentration of paraga and assess paraga peddlers’ knowledge of paraga composition, production and usage [11]. This is the second part, which is to determine the prevalence and pattern of paraga use among intercity commercial drivers.

## Methods

Data Sources: The survey was conducted in Osogbo, the capital of Osun State in southwest Nigeria. It is a nodal town with road connections to surrounding towns and other cities in the country. The Yoruba, one of Nigeria’s three major ethnic groups, is the predominant ethnic group in this part of the country. The others are the Hausa/Fulani in the north and the Igbo in the southeast. Also in Nigeria, the first and second levels of education are the primary school education and the secondary school education, both of which have durations of six years. Candidates with good grades at the school leaving examination at this stage are eligible to proceed to the post-secondary level at colleges of education, polytechnics or universities with duration ranging from three to six years. The two major religions in Nigeria are Christianity and Islam.

The target population was the intercity commercial drivers operating from the motor parks in Osogbo. Commercial drivers belong to the National Union of Road Transport Workers (NURTW), which has an organization structure made up of a state chairman and other officers. The smallest units are the motor parks, each with its unit chairman. Osogbo has seven of these units and they were the locations of the survey. Each motor park consists of a large open space surrounded by kiosks and shops where food stuffs, drinks including herbal mixtures, and other items are sold.

The aim was to determine the prevalence of paraga use among intercity commercial drivers in Osogbo. The level of awareness of paraga use was also assessed and socio-economic correlates of paraga use were investigated

A questionnaire consisting of two parts was used to interview the drivers. The first part consisted of questions about respondents’ personal data while the second part was based on the WHO’s methodology for drug survey, which has been previously validated in Nigeria [12-14]. It appraised the pattern of paraga use, factors associated with its use; and the level of awareness of the consequences of paraga consumption by the respondents. Current or active users were defined as those who had taken the substance at least once in the past 30 days [12].

Ethical consideration*:* Ethical permission was obtained from the ethical committee of the Ladoke Akintola University of Technology Teaching Hospital Osogbo. Permission was also obtained from the state chairman of NURTW. A written consent was included on the first page of the questionnaire. The drivers were encouraged to fill the forms themselves, but two trained research assistants were recruited to administer the questionnaires to those who did not understand written or spoken English.

Sample size calculation was based on the 67.2% prevalence rate of alcohol consumption among commercial drivers in south-west Nigeria [14]. Subjects were recruited from all the motor parks based on proportional sampling technique [15]. All drivers registered with each unit were eligible for inclusion. Data was analyzed using SPSS 15.

## Results

Three hundred and fifty questionnaires were distributed and collected, but 18 (5.4%) had too many missing data and were rejected, leaving 332 (94.6%) for the final analysis. All respondents were males. Table 1 shows other sociodemographic characteristics of the drivers.

**Table 1:** Sociodemographic and some other characteristics of the drivers

**Attribute**	**Number (%)**
Age	
Mean Age	40.6 years
Standard deviation	9.1 years
Marital status (N = 220)	
Married	229 (71.5)
Single	45 (14.1)
Widowed	49 (14.4)
Religion (N = 316)	
Islam	182 (57.6)
Christian	134 (42.4)
Education (N = 300)	
None	8 (2.7)
Primary	109 (36.3)
Secondary	155 (51.7)
Post-secondary	28 (9.3)
Ethnicity	
Yoruba	284 (89.3)
Igbo	26 (8.2)
Hausa	8 (2.5)

The mean driving experience was 14.3 (SD 7.1) years. The median distance travelled per day was 120 Km, ranging between 10 and 715 Km. Thirty-five (10.7%) drivers averaged one trip per day, 139 (42.6%) averaged two trips per day, 108 (33.1%) averaged three and 44 (13.5%) averaged four trips per day. *Paraga Use*.

Paraga use was popular among drivers (Table 2). Most were consistent users; for example, the difference between lifetime prevalence and prevalence in the past one year was just 3.0%. Most respondents were heavy or moderate users, and most either use it before or during work, or with no regard to driving activities (Table 3). One hundred and sixty-two (48.8%) respondents felt it was very/fairly easy to get paraga, while only 30 (9.0%) thought it was difficult and 12 (3.6%) felt it was probably impossible. Of the 184 respondents who disclosed where they obtained paraga from, 164 (89.1%) were getting it either in the motor parks or very close to the parks. The remaining 20 (11.9%) obtained paraga either at home or in their friends’ place.

**Table 2:** Measures of paraga use among drivers

**Measure of paraga use**	**Number of users (%)**
Lifetime Prevalence Rate (Ever use)	188 (56.6)
Prevalence Rate in the past one year (Past year use)	178 (53.6)
Prevalence Rate in the past one month (Past one month)	160 (48.2)
Prevalence rate for today	136 (29.5)

**Table 3:** Pattern of alcohol use among current users of paraga

**Variable**	**Number of respondents (%)**
Extent of use (n = 160)	
Light Use (1–5 days)	40 (25.0)
Moderate use (6–19 days)	82 (51.4)
Heavy use (>20 days)	38 (23.6)
Time of use (n-160)	
In the morning before work	16 (10.0)
During work	28 (17.5)
After work	72 (45.0)
At any time of the day	44 (27.5)

To avoid recollection bias, this questions were directed only to the 160 drivers who were current users of paraga.

There were no significant differences in the mean age, mean driving experience and mean distance travelled per day between drivers who were current users and those who were not.

### Drivers’ knowledge of effects of paraga

When asked about whether paraga was harmful or beneficial; 200 (60.2%) drivers responded. Eighty (40.0%) thought that paraga was harmful to users, 50 (25.0%) believed paraga was beneficial while the remaining 70 (35.0%) were undecided about the harmful effects of paraga. Fifty seven of the 80 drivers who thought paraga was harmful suggested what the harmful effects were. Most (18, 31.6%) opined that paraga can cause people to behave badly, 13 (22.8%) believed it was harmful because it could contribute to vehicle crashes, 12 (21.1%) and 10 (17.5%) believed that paraga could cause liver and kidney diseases respectively. Three (5.3%) were of the opinion that they could cause other unspecified physical illnesses while one thought taking it can lead to family separation.

When asked to list the benefits that could be derived from paraga, 86 drivers responded to this question. These 86 respondents included not only 48 of the original 50 respondents who believed paraga to be beneficial, but also 36 of those who felt paraga to be harmful and 2 of those who were not sure about the effects of paraga. Table 4 showed that after back pain, most of the imagined benefits were social (energizes and emboldens, enhancing libido and helping to cope with frustration). When asked to give their opinions about how dangerous it was to drive while under the influence of paraga, 48 (14.5%) thought it was very dangerous, 32 (9.6%) thought it was quite dangerous, 56 (16.9%) thought it was not very dangerous while 14 (4.2%) thought it was not dangerous and 54 (16.3%) were not sure.

**Table 4:** Believes and practices of drivers regarding paraga

**Variable**	**Number of respondents (%)**
Suggested benefits of paraga (n = 131)	
Treats back pain	55 (42.0)
Energizes and emboldens and keeps alert	37 (28.2)
Increases libido	17 (12.9)
Helps to cope with frustration	8 (6.1)
Treat piles	6 (4.6)
Improves appetite	4 (3.1)
Treats diseases	4 (3.1)
Reasons for taking paraga (n = 193)	
Taste is enjoyable	28 (14.5)
To ward-off cold	62 (32.2)
Treat illnesses	44 (22.8)
For more energy	35 (18.1)
To be like others	12 (6.2)
To keep alert	12 (6.2)

**Table 5:** Perceived harmfulness of paraga use and willingness to stop taking it among current users

**Perceived harmfulness**	**Willingness to stop**			**Total**
	**Yes (%)**	**No (%)**	**Not sure (%)**	
Not harmful	16 (36.4)	20 (45.5)	8 (18.2)	44
Harmful	24 (37.5)	26 (40.6)	14 (21.9)	64
Don’t know	28 (56.0)	2 (4.0)	20 (40.0)	50
Total	68 (43.0)	48 (30.4)	42 (26.6)	158

### Drivers’ attitude and practice of taking paraga

One hundred and eighty four (55.4%) drivers admitted to driving after consuming paraga in the past. Of these, 42 (22.8%) were doing it on most days, 96 (52.2%) between 1–5 times a week, 18 (9.8%) at least once a month and 28 (15.2%) rarely. One hundred and twenty-two (36.7%) had actually seen someone behave drunkenly after consuming paraga and 88 (26.5%) admitted to having been involved in a road crash in the past after taking paraga. Finally, we asked current (past one month) users about their willingness to stop taking paraga, 48 (30.0%) said they were not willing to stop, 70 respondents (43.8%) said were willing to stop, and 42 (26.3%) did not think that taking paraga was a problem. When willingness to stop taking paraga was compared to driver’s perceived harmfulness of paraga, only 37.5% of those who felt paraga to be harmful would be willing to stop it, while two-fifth would not be willing (Table 5).

## Discussion

This study showed that paraga is commonly consumed by commercial vehicle drivers operating out of the motor parks in Osogbo, southwest Nigeria. The lifetime use by 55.6% of the drivers is higher than the 51.6% recorded among secondary school students in Lagos [7]. This is worrisome because almost all the drivers (184 out of 188 drivers) who admitted to taking paraga in the past also admitted to driving shortly after consuming paraga in the past, in fact close to 75% of these respondents were doing it at least once a week. Paraga is basically an alcoholic drink masquerading as a medicinal preparation; it is therefore a major public health problem if a sizable proportion of commercial drivers consistently drive under its influence [7,8].

Furthermore, paraga drinkers appear to be consistent in the usage of the drink: the percentage difference between lifetime and current use prevalence is less than 10%. This is similar to the findings of Oshodi et al. who also reported a less than 10% point difference between lifetime and current use among secondary school students in Lagos [7]. Similarly, Kadiri in a study of paraga use in a community in Lagos showed that 60% of paraga users were taking it on daily basis [16]. Looking at the reasons given by the drivers for taking paraga offers some explanations for this consistency in usage. For 55% of the drivers, the reason was to treat common cold and other illnesses. Thus, the respondents might continue to take the concoction as long as they felt they still had the illness for which paraga was being used. They might keep on taking it, even when they know it might be harmful; equating any harmful effects to the side effects of conventional drugs. This may explain the reason why 45% of those who felt that paraga was harmful also deemed paraga useful for treating illnesses. In addition, the relative high cost of orthodox medicine might have encouraged the use of alternative medicine among the populace [7]. The persistent use might also be because the drivers were getting addicted to some of the constituents of paraga; the most likely candidate being alcohol. Reasons such as “enjoyable taste”, “makes me to become more alert” and “makes me gain more energy” might be the manifestations of alcohol dependence [17].

It is also instructive that almost all the suggested benefits were for treating chronic illnesses like back pain or coping with emotional and stress related conditions. These are conditions that are very difficult to manage, for example, chronic back pain, which is an occupational disease of drivers, can be very difficult to treat satisfactorily and thus, patients may be encouraged to try different types of treatment [18].

Three quarters of current users were moderate to heavy users. Also, more than 50% of current users take paraga before driving, while driving, or at any time of the day, meaning that a sizable proportion of the drivers were at risk of driving under influence. This fact is buttressed by the admission by 25.6% of the drivers that they have been involved in road crashes when they drove after taking paraga. In addition, more than one-thirds of the drivers had seen someone become drunk on paraga.

We have shown that about 56. 3% of current users were either not willing to stop taking paraga, or they did not see any problem attached to paraga intake. How can these persons be persuaded to stop taking the concoction? Trying to address this question brings another one: Is there even a need for them to stop? Should the focus be on persuading them to avoid paraga if they want to drive?

We have established in paper one of this study, that paraga contains alcohol concentration that is similar to alcoholic drinks [11,19]. It may therefore, be risky to drive after taking paraga, and on this evidence alone, drivers should be advised against driving after consuming alcohol. However, paraga also contains other ingredients such as herbs and minerals in concentrations that can fluctuate because the production is not controlled [7,16,19]. This can be dangerous because most medicines, including herbs are potentially dangerous when used inappropriately or in excess [20]. Furthermore, combining herbs or taking them without other herbs that are traditionally used to ameliorate their side effects can cause serious harms [21]. Traditionally in Africa, diseases are treated with herbs prescribed by traditional practitioners whose knowledge had been passed down through generations [6]. These medicine men had the required ethnobotanical knowledge to correctly combine the herbs. However, urbanization and modernization has broken down this process, and herbs are now commonly purveyed on the street. Paraga is one of the most popular forms in which herbal medicines are now dispensed on the street, and this popularity has come with a cost as there have been newspaper reports of deaths following consumption of paraga [22,23]. The production and distribution of paraga needs to come under the scrutiny of the National Agency for Food and Drug Administration and Control (NAFDAC), which has the responsibility for controlling and regulating the manufacture, sale and packaging of food and drug in Nigeria. There is a vital need to study the photochemistry of the herbs used in formulating paraga, and to establish their efficacy. This will provide a scientific basis for establishing a policy on paraga use among the populace. In the meantime, drivers need to be educated on the potential of paraga to increase the risk for alcohol related traffic injuries and to reduce access to the concoction by restricting its sales in motor parks. Commercial drivers in Nigeria are members of the National Union of Road Transport Workers (NURTW) the Road Transport Employers Association of Nigeria (RTEAN) [24,25]. These unions have a powerful hold on their members [8], and this has been capitalized-on by the authorities to successfully promote certain public health issues such as HIV/AIDS control and Accident Insurance among drivers [24,25]. Such an approach can also be used to control paraga and other alcoholic drink consumption use by commercial drivers.

## Conclusions

Commercial drivers operating from motor parks in Osogbo have a high prevalence rate for paraga use. While the risk/benefit to health is not yet fully established, the alcoholic nature of the drinks has been proven. Therefore, drivers’ access to the concoction should be controlled and appropriate enforcement put in place.

## Competing interests

The authors declare that they have no competing interests.

## Authors’ contributions

OSK designed the study, executed the data management, and drafted the manuscript. FFO provided the validated questionnaire, helped in the conceptualization and revised the manuscript. Both authors read and approved the final manuscript.
